# Celery seed extract attenuates sarcopenic obesity and age-related sarcopenia by reducing intramuscular lipid accumulation in mice

**DOI:** 10.3389/fnut.2026.1861420

**Published:** 2026-09-01

**Authors:** Su-Kyung Shin, Jiwon Jeong, Min-Jeong Kim, Mi Jin Jang, Chae Lee Park, Sunhoo Kim, Eun-Young Kwon

**Affiliations:** 1 Department of Food Science and Nutrition, Kyungpook National University, Daegu, Republic of Korea; 2 Center for Food and Nutritional Genomics Research, Kyungpook National University, Daegu, Republic of Korea; 3 Department of Global Innovative Drugs, The Graduate School of Chung-Ang University, Seoul, Republic of Korea; 4 Preclinical Research Center, Daegu-Gyeongbuk Medical Innovation Foundation, Daegu, Republic of Korea; 5 Huons Dongam R&D Center, HuonsN, Seongnam, Gyeonggi, Republic of Korea; 6 Center for Beautiful Aging, Kyungpook National University, Daegu, Republic of Korea; 7 Department of Advanced Bioconvergence, Kyungpook National University, Daegu, Republic of Korea

**Keywords:** AMPK–PGC-1α, celery seed extract, intramuscular lipid accumulation, mitochondrial metabolism, NLRP3 inflammasome, sarcopenia

## Abstract

**Background & aims:**

Sarcopenia is characterized by progressive loss of skeletal muscle mass and function and is increasingly recognized to be influenced by metabolic disturbances associated with aging and obesity. Intramuscular lipid accumulation has emerged as a key pathological feature linking metabolic dysfunction to skeletal muscle deterioration. Celery seed extract (CSE) possesses anti-obesity, anti-inflammatory, and antioxidant properties; however, its potential role in skeletal muscle metabolism has not been well investigated. This study aimed to determine whether CSE attenuates skeletal muscle deterioration associated with obesity and aging through modulation of intramuscular lipid accumulation and related metabolic pathways.

**Methods:**

Diet-induced obese mice and naturally aged mice were used to evaluate the effects of CSE supplementation. Skeletal muscle mass, grip strength, muscle morphology, intramuscular lipid content, mitochondrial metabolic signaling, inflammatory responses, and muscle protein turnover pathways were assessed using biochemical, molecular, and histological analyses.

**Results:**

CSE supplementation significantly improved skeletal muscle mass, grip strength, and muscle fiber cross-sectional area in both obese and aged mice. These improvements were accompanied by reduced intramuscular triglyceride and cholesterol accumulation. Mechanistically, CSE improved mitochondrial metabolic signaling by activating the AMPK–PGC-1α pathway and increasing mitochondrial oxidative phosphorylation proteins. In addition, CSE suppressed inflammatory signaling pathways, including MAPK activation and NLRP3 inflammasome signaling, and improved muscle proteostasis by enhancing myogenic regulators while reducing the expression of proteolytic factors such as MuRF1, Atrogin-1, and myostatin. Correlation analyses further indicated that intramuscular lipid accumulation was closely associated with mitochondrial dysfunction, inflammatory activation, and muscle atrophy.

**Conclusions:**

These findings demonstrate that CSE alleviates skeletal muscle deterioration in both obesity- and aging-associated sarcopenia by reducing intramuscular lipid accumulation and improving mitochondrial metabolism, inflammatory responses, and muscle protein turnover. Targeting intramuscular lipid accumulation may therefore represent a promising nutritional strategy for preventing sarcopenia associated with metabolic and aging-related stress.

## 1 Introduction

Sarcopenia is a progressive and generalized skeletal muscle disorder characterized by the loss of muscle mass and function, including declines in muscle strength and physical performance. The European Working Group on Sarcopenia in Older People (EWGSOP) defines sarcopenia as low muscle mass accompanied by impaired muscle strength or reduced physical performance, reflecting both structural and functional deterioration of skeletal muscle (1). Sarcopenia has become a major clinical concern in aging societies because it is strongly associated with adverse outcomes such as disability, impaired mobility, increased risk of falls, loss of independence, and mortality (1). Epidemiological studies indicate that its prevalence increases markedly with age, affecting a substantial proportion of individuals over 60 years and imposing a significant burden on public health systems (2). Consequently, understanding the mechanisms underlying sarcopenia and identifying effective strategies to prevent or attenuate skeletal muscle loss have become important priorities in clinical nutrition and metabolic research.

Although sarcopenia has traditionally been regarded as an age-related disorder, accumulating evidence indicates that obesity also contributes to skeletal muscle deterioration. The coexistence of excessive adiposity and reduced muscle mass and function, termed sarcopenic obesity, is associated with greater risks of metabolic abnormalities, functional impairment, disability, and mortality than either condition alone (2). Obesity-induced adipose tissue dysfunction, characterized by adipocyte hypertrophy, immune cell infiltration, and dysregulated adipokine secretion, promotes chronic low-grade inflammation that negatively affects skeletal muscle metabolism (3). In addition, excess fatty acids released from adipose tissue can accumulate ectopically within skeletal muscle as intramyocellular lipids, leading to lipotoxicity, mitochondrial dysfunction, insulin resistance, and inflammatory signaling that contribute to muscle atrophy and impaired muscle performance (3).

Both aging and obesity promote ectopic lipid deposition within skeletal muscle, a process commonly referred to as intramuscular lipid accumulation. Intramuscular lipid accumulation involves lipid accumulation within muscle fibers as well as adipose infiltration between and within muscle bundles, resulting in alterations in muscle composition and metabolic function (4). Increased fat infiltration in skeletal muscle has been associated with reduced muscle quality, impaired mobility, and increased risk of functional decline in older adults (5). Mechanistically, excessive lipid accumulation induces lipotoxic stress, mitochondrial dysfunction, and chronic low-grade inflammation, thereby disrupting metabolic homeostasis and impairing oxidative metabolism (6). Dysregulated fatty acid transport and impaired mitochondrial fatty acid oxidation further contribute to inflammatory signaling and metabolic dysfunction in skeletal muscle (7). Collectively, these findings indicate that intramuscular lipid accumulation represents a critical pathological link between metabolic dysfunction and skeletal muscle deterioration.

Intramuscular lipid accumulation can disrupt key metabolic pathways that regulate skeletal muscle homeostasis. In particular, impairment of the AMP-activated protein kinase (AMPK)–peroxisome proliferator-activated receptor gamma coactivator 1-alpha (PGC-1α) signaling axis, a central regulator of mitochondrial biogenesis and oxidative metabolism, has been implicated in skeletal muscle dysfunction and atrophy (8, 9). Reduced activity of this pathway has been associated with mitochondrial dysfunction and impaired oxidative metabolism in both aging and obesity (10). Human studies have demonstrated age-related declines in mitochondrial DNA abundance, oxidative enzyme activity, and ATP production in skeletal muscle (11). Lipid accumulation can also promote chronic low-grade inflammation through activation of innate immune signaling pathways such as the NOD-like receptor family pyrin domain-containing 3 (NLRP3) inflammasome, contributing to metabolic dysfunction (12). Elevated inflammatory cytokines, including interleukin (IL)-6, have been associated with increased risk of functional decline in older adults and can directly induce skeletal muscle atrophy (13, 14). Chronic inflammatory signaling further promotes activation of catabolic pathways, such as the ubiquitin–proteasome system, and increases expression of atrophy-related genes, including atrogin-1 and muscle RING finger-1 (MuRF1), ultimately leading to enhanced proteolysis and muscle wasting (15, 16).

Celery (*Apium graveolens* L.), a member of the Apiaceae family, has long been used in traditional medicine and has recently attracted attention because of its diverse biological activities. Celery contains various phytochemicals, including flavonoids, phenolic acids, and essential oil components, which contribute to its antioxidant and therapeutic properties (17). CSE has been reported to exert beneficial metabolic effects, including suppression of adipogenesis and lipid accumulation in adipocytes and anti-inflammatory activity through inhibition of inflammatory mediators (18, 19). Celery preparations have also been traditionally used for inflammatory conditions such as arthritis, and celery-derived phytochemicals exhibit strong antioxidant activity (17, 20). Emerging evidence further suggests that celery supplementation may improve cardiometabolic parameters in individuals with metabolic abnormalities (21). Despite these promising properties, the impact of CSE on skeletal muscle metabolism and muscle deterioration remains largely unexplored. In particular, whether CSE can modulate intramuscular lipid accumulation and the associated metabolic and inflammatory pathways that contribute to muscle atrophy remains unclear.

Therefore, the present study aimed to investigate whether CSE can attenuate skeletal muscle deterioration associated with both obesity and aging. Using diet-induced obese mice and naturally aged mice, we examined whether CSE supplementation reduces intramuscular lipid accumulation and improves skeletal muscle metabolic homeostasis. In particular, this study evaluated whether CSE modulates key molecular pathways involved in muscle metabolism and atrophy, including the AMPK–PGC-1α signaling axis, mitochondrial oxidative metabolism, inflammatory responses, and proteolytic pathways in skeletal muscle. By identifying shared metabolic mechanisms underlying obesity- and age-related muscle dysfunction, this study sought to determine whether CSE may represent a potential nutritional strategy for ameliorating sarcopenia driven by common metabolic disturbances.

## 2 Material and methods

### 2.1 Preparation of CSE

Celery seeds (Apium graveolens L.) were obtained as dried whole seeds from AB Mauri India Pvt. Ltd., and the CSE used in this study was provided by HuonsN (Seongnam, Korea). The seeds were extracted twice with 30% (v/v) ethanol using a 10-fold solvent-to-sample ratio (v/w) at 90 ± 5 °C for 6 h under controlled heating conditions. The resulting extract was filtered through a 50-mesh filter to remove insoluble materials. The filtrate was concentrated under reduced pressure at 50–70 °C until the soluble solid content reached 25–30 Bx, as determined using a Brix refractometer. The concentrated extract was subsequently spray-dried using dextrin as a carrier, yielding approximately 30 ± 5% dry extract powder, which was used for subsequent experiments. The extraction process was standardized based on extraction yield and HPLC analysis using Graveobioside A, a flavone glycoside present at 19.12 mg/g of CSE, as the quantitative marker compound to ensure batch-to-batch consistency.

### 2.2 Animal experiments

Male C57BL/6J mice (6 weeks old) were purchased from Central Lab Animal Inc. (Seoul, Korea). Animals were maintained under controlled environmental conditions (temperature 25 ± 2 °C, relative humidity 40–60%, and a 12 h light/dark cycle) with free access to food and water. After a one-week acclimation period with a standard laboratory chow diet, mice were randomly assigned to experimental groups. Mice were fed either a normal diet (ND, 10% kcal from fat) or a high-fat diet (HFD, 40% kcal from fat) throughout the experimental period. The detailed composition of the experimental diets is provided in Supplementary Table S1.

No formal sample size calculation was performed. Sample sizes were determined based on previous studies employing similar experimental designs. For the diet-induced obesity model, a total of 46 mice were randomly divided into six groups: ND (*n* = 8), HFD (*n* = 8), HFD supplemented with celery seed extract at 50 mg/kg body weight (CSE50, *n* = 8), 100 mg/kg body weight (CSE100, *n* = 8), or 200 mg/kg body weight (CSE200, *n* = 8), and a positive control group receiving orlistat (20 mg/kg body weight, *n* = 6). CSE was provided by HuonsN Co. (Seongnam, Korea). CSE and orlistat were freshly suspended in distilled water and administered once daily by oral gavage for 12 weeks. The ND and HFD groups received an equivalent volume of distilled water as a vehicle.

To evaluate the effects of CSE on age-associated muscle decline, a middle-aged mouse model was also established. Male C57BL/6J mice aged 7 weeks (young control, YC, *n* = 6) and 55–56 weeks (aged control, AC, *n* = 6) were acclimated for 1 week and maintained on a normal chow diet containing 10 kcal% fat. Middle-aged mice were randomly assigned to receive CSE at doses of 50 mg/kg (CSE50, *n* = 6), 100 mg/kg (CSE100, *n* = 6), or 200 mg/kg body weight (CSE200, *n* = 6), or oxymetholone (OXM, 50 mg/kg body weight, *n* = 6) as a positive control for muscle strength improvement. Treatments were administered orally once daily for 12 weeks. The selected dose range was based on previous clinical and toxicological studies of celery seed extract (22, 23), and the 12-week intervention period was selected based on previous chronic HFD-induced obesity studies (24).

At the end of the experimental period, mice were fasted for 14 h and anesthetized with isoflurane. Blood samples were collected from the abdominal aorta using heparinized syringes and centrifuged at 1,800 × g for 10 min at 4 °C to obtain plasma. Tissues, including skeletal muscle and adipose tissue, were rinsed with cold saline, weighed, rapidly frozen in liquid nitrogen, and stored at −70 °C until further analysis.

All animal procedures were conducted in accordance with the guidelines for the care and use of laboratory animals and were approved by the Institutional Animal Care and Use Committee (IACUC) of the Preclinical Research Center, Daegu Gyeongbuk Medical Innovation Foundation (Approval Nos. KMEDI-23121404-00 and KMEDI-24041101-00). This study was conducted and reported in accordance with the ARRIVE 2.0 guidelines.

### 2.3 Grip strength and muscle thickness

Forelimb grip strength was measured using a digital force gauge (JM Instruments Corp., Seoul, Korea). Before measurement, mice were acclimated to the grip strength apparatus for five consecutive days to minimize stress and variability. Grip strength was assessed at week 12 of the experimental period. Each mouse was allowed to grasp the metal grid attached to the force gauge, and the peak pulling force was recorded. The measurement was repeated three times for each mouse, and the mean value was calculated and normalized to body weight (N/BW).

Hindlimb muscle thickness was measured at week 12 using a digital caliper (Goyang, Korea). The thickness of the hindlimb was measured and expressed relative to body weight (mm/g BW).

### 2.4 Plasma TNFα and IL-6

Plasma levels of tumor necrosis factor-α (TNF-α) and IL-6 were quantified using a Mouse Focused Multi-Plex Discovery Assay Kit (MilliporeSigma, MA, USA) according to the manufacturer's protocol.

### 2.5 Histological analysis

Skeletal muscle tissues were fixed in 10% neutral buffered formalin and subsequently embedded in paraffin. Paraffin-embedded samples were sectioned at a thickness of 5 μm and mounted on poly-L-lysine–coated slides. Tissue sections were stained with hematoxylin and eosin (H&E) to evaluate muscle morphology and Sirius Red to assess collagen deposition. For immunohistochemical analysis, muscle sections were incubated with antibodies against insulin-like growth factor-1 (IGF-1) and myostatin. Details of the antibodies used for immunohistochemistry are provided in Supplementary Table S2. Stained sections were examined using an optical microscope (Zeiss Axio Scope, Oberkochen, Germany) at magnifications of 200 × or 400 × .

### 2.6 Muscle protein and intramuscular lipids

Intramuscular lipids were extracted from skeletal muscle tissues using a modified method based on the Folch procedure (25). Briefly, muscle samples were homogenized, and lipids were extracted using organic solvents. The dried lipid extracts were reconstituted in ethanol, followed by the addition of an emulsifying solution containing Triton X-100 and sodium cholate. Triglyceride (TG) and total cholesterol (TC) concentrations were subsequently quantified using enzymatic colorimetric assay kits (Asan Pharmaceutical Co., Seoul, Korea) according to the manufacturer's instructions.

For muscle protein quantification, skeletal muscle tissues were homogenized in 200–300 μl of lysis buffer and centrifuged at 22,000 × g RCF for 20 min at 4 °C. The supernatant was collected, and total protein concentration was determined using a Pierce BCA Protein Assay Kit (Thermo Scientific, MA, USA). Protein levels were calculated based on a standard curve generated with bovine serum albumin (BSA).

### 2.7 Western blotting

Skeletal muscle tissues were homogenized in lysis buffer, and the lysates were centrifuged to obtain the protein-containing supernatant. Protein concentrations were determined using a Pierce BCA Protein Assay Kit (Thermo Fisher Scientific, MA, USA). Equal amounts of protein (20–30 μg) were separated by electrophoresis on 10% SDS–polyacrylamide gels and subsequently transferred onto polyvinylidene difluoride (PVDF) membranes (Merck Millipore, MA, USA).

Membranes were briefly stained with Ponceau S to confirm equal protein transfer and then blocked with 5% skim milk (Kisanbio Co., Seoul, Korea) for 1 h at room temperature. The membranes were incubated overnight at 4 °C with primary antibodies, followed by washing with TBS containing 0.1% Tween-20 (TBST). Subsequently, membranes were incubated with horseradish peroxidase (HRP)-conjugated secondary antibodies for 1 h at room temperature.

Protein bands were visualized using an enhanced chemiluminescence detection kit (WESTAR ECL, Cyanagen, Bologna, Italy) and imaged using a Fusion evo-6 imaging system (Vilber, Collégien, France). Band intensities were quantified using the accompanying software and normalized to GAPDH as a loading control. Details of the antibodies used in this study are provided in Supplementary Table 2.

### 2.8 Real-Time quantitative PCR (RT-qPCR)

Total RNA was isolated from skeletal muscle tissues using TRIzol reagent (Invitrogen, MA, USA) according to the manufacturer's instructions. Following chloroform extraction, RNA was precipitated with isopropanol, washed with 70–75% ethanol, and dissolved in RNase-free water. RNA concentration and purity were determined using a NanoDrop spectrophotometer (Thermo Fisher Scientific, MA, USA), and samples were adjusted to a concentration of approximately 500 ng/μl.

Complementary DNA (cDNA) was synthesized from total RNA using the PrimeScript™ RT Reagent Kit with gDNA Eraser (Takara Bio Inc., Shiga, Japan). RT-qPCR was performed using SYBR Green Master Mix (Enzynomics Co., Daejeon, Korea) in a total reaction volume of 20 μl containing SYBR Green solution, cDNA template (2 μl), and gene-specific primers (200 nM each).

Amplification was conducted using a CFX Duet Real-Time PCR System (Bio-Rad, CA, USA) under the following cycling conditions: initial denaturation at 95 °C for 10 min, followed by 40 cycles of denaturation at 95 °C for 10 s, annealing at 60 °C for 15 s, and extension at 72 °C for 30 s. Relative gene expression levels were calculated using the comparative cycle threshold (2^∧^-ΔΔCt) method, with GAPDH used as the internal reference gene. Primer sequences used in this study are listed in Supplementary Table 3.

### 2.9 Transcriptome sequencing and gene set enrichment analysis

Transcriptomic profiling was performed using liver and epididymal white adipose tissues obtained from the high-fat diet-induced obesity model. Total RNA was extracted and used for library preparation with the TruSeq Stranded mRNA Sample Preparation Kit (Illumina, CA, USA), followed by paired-end sequencing on the Illumina NovaSeq X platform (Macrogen Inc., Seoul, Korea). Differential gene expression analysis was performed using DESeq2 (adjusted *P* < 0.05), followed by gene set enrichment analysis (GSEA) using multiple pathway databases, including MSigDB, KEGG, Reactome, Gene Ontology Biological Process, and WikiPathways. Representative enriched pathways relevant to amino acid metabolism and muscle-related biological processes are presented in Supplementary Figure S1.

### 2.10 Statistics

All data are presented as mean ± standard error of the mean (SEM). Statistical analyses were performed using SPSS software (version 29.0; IBM SPSS Statistics, Chicago, IL, USA). Differences between the ND and HFD groups in the obesity model and between the YC and AC groups in the aging model were evaluated using Student's *t*-test. Differences among treatment groups within each model were analyzed using one-way analysis of variance (ANOVA) followed by Duncan's multiple range test. Statistical significance was defined as *p*<*0.05*.

## 3 Results

### 3.1 CSE ameliorates obesity- and aging-induced sarcopenia

**Figure 1:**
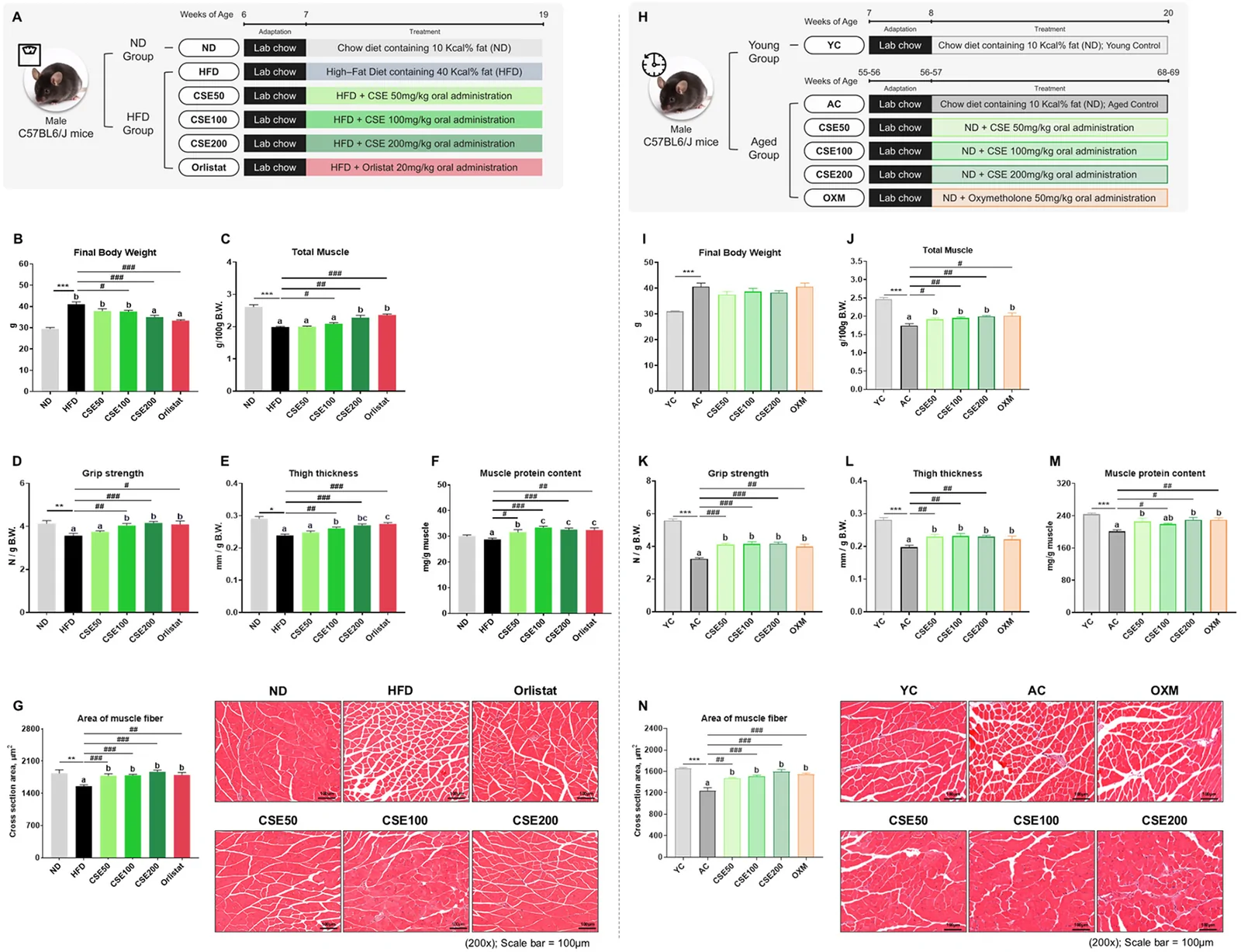
**Celery seed extract attenuates skeletal muscle loss and functional decline in obese and aged mice. (A, H)** Experimental design of the obesity-induced and aging-associated sarcopenia models. **(B, I)** Final body weight in obese and aged mice. **(C, J)** Total skeletal muscle weight. **(D, K)** Grip strength. **(E, L)** Thigh thickness. **(F, M)** Muscle protein content. **(G, N)** Representative hematoxylin and eosin **(H&E)**-stained images of skeletal muscle and quantification of muscle fiber cross-sectional area. Data are presented as mean ± SEM (*n* = 6–8). *P < *0.05*, **P < *0.01*, ***P < *0.001* versus ND or YC; ^#^*P* < *0.05*, ^*##*^*P* < *0.01*, ^*###*^*P* < *0.001* versus HFD or AC. ND, normal diet; HFD, high-fat diet; CSE, celery seed extract (50, 100, or 200 mg/kg body weight); Orlistat, 20 mg/kg body weight; YC, young control; AC, aged control; OXM, oxymetholone (50 mg/kg body weight). Different letters (a–c) indicate significant differences among the HFD or aged groups at *P* < *0.05*. Total muscle weight, Quadriceps + Tibialis anterior + Gastrocnemius + Soleus.

The experimental designs for the diet-induced obesity model and the aging model are illustrated in Figures 1A, 1H, respectively. In the obesity model, male C57BL/6J mice were fed either an ND or an HFD, and HFD-fed mice received celery seed extract (CSE; 50, 100, or 200 mg/kg) or orlistat as a reference treatment. As expected, HFD feeding significantly increased final body weight compared with ND controls (Figure 1B). Although CSE treatment produced modest reductions in body weight relative to the HFD group, the effect was less pronounced than that observed with orlistat.

Despite the increased body weight, HFD feeding resulted in a significant reduction in skeletal muscle mass and functional performance. Total skeletal muscle mass was significantly decreased in HFD-fed mice compared with ND controls (Figure 1C), accompanied by impaired grip strength (Figure 1D), decreased thigh thickness (Figure 1E), and reduced muscle protein content (Figure 1F). Administration of CSE significantly improved these parameters. Compared with the HFD group, CSE-treated mice exhibited increased total muscle mass, enhanced grip strength, greater thigh thickness, and higher muscle protein content. Histological examination further supported these findings, showing that HFD feeding reduced the cross-sectional area of muscle fibers, whereas CSE treatment restored muscle fiber size toward levels observed in ND controls (Figure 1G).

To further characterize the effects of CSE on skeletal muscle, individual muscle weights were analyzed (Table 1). Consistent with the reduction in total muscle mass, HFD feeding significantly decreased the weights of several individual muscles, including the quadriceps, tibialis anterior, and gastrocnemius muscles, compared with ND controls. CSE treatment attenuated these reductions in a dose-dependent manner. In particular, CSE200 significantly restored quadriceps and gastrocnemius muscle mass relative to the HFD group, reaching levels comparable to those observed with orlistat treatment. Similar trends were observed in the tibialis anterior muscle, whereas the soleus muscle showed relatively modest changes across groups. These results indicate that CSE alleviates obesity-associated skeletal muscle loss across multiple muscle groups.

**Table 1:** Effects of celery seed extract on skeletal muscle mass in obese mice.

g per 100 g body weight	ND	HFD	CSE50	CSE100	CSE200	Orlistat
**Quadriceps**	1.10 ± 0.03	0.81 ± 0.01^a***^	0.81 ± 0.02^**a**^	0.83 ± 0.02^**a**^	0.92 ± 0.04^**b##**^	0.93 ± 0.03^**b#**^
**Tibialis anterior**	0.48 ± 0.02	0.37 ± 0.01^a***^	0.38 ± 0.01^**ab**^	0.38 ± 0.01^**ab**^	0.42 ± 0.02^**bc**^	0.44 ± 0.01^**c###**^
**Gastrocnemius**	1.00 ± 0.02	0.76 ± 0.01^a***^	0.79 ± 0.01^**ab**^	0.82 ± 0.01^**b##**^	0.90 ± 0.02^**c###**^	0.94 ± 0.01^**c###**^
**Soleus**	0.059 ± 0.002	0.049 ± 0.002^**^	0.046 ± 0.002	0.051 ± 0.002	0.052 ± 0.002	0.052 ± 0.003

Skeletal muscle mass is expressed as g per 100 g body weight. Data are presented as mean ± SEM (n = 6–8). ^**^P < 0.01, ^***^P < 0.001 versus ND; ^#^*P* < 0.05, ^##^*P* < 0.01, ^###^*P* < 0.001 versus HFD. ND, normal diet; HFD, high-fat diet; CSE, celery seed extract (50, 100, or 200 mg/kg body weight); Orlistat, 20 mg/kg body weight. Different letters (a–c) indicate significant differences among the HFD-fed groups at *P* < 0.05.

To determine whether the beneficial effects of CSE extend to age-related muscle deterioration, an aging model was subsequently evaluated (Figure 1H). Compared with YC mice, AC mice exhibited significantly higher body weight (Figure 1I) together with marked reductions in total muscle mass (Figure 1J), grip strength (Figure 1K), thigh thickness (Figure 1L), and muscle protein content (Figure 1M), consistent with age-associated muscle decline. CSE treatment significantly improved these muscle parameters in aged mice. Relative to the AC group, CSE administration increased muscle mass, grip strength, thigh thickness, and muscle protein content. Consistent with these functional improvements, histological analysis revealed that aging markedly reduced muscle fiber cross-sectional area, whereas CSE treatment partially restored muscle fiber morphology (Figure 1N).

Analysis of individual muscles further confirmed these effects (Table 2). Compared with YC mice, AC mice displayed significant reductions in quadriceps, tibialis anterior, gastrocnemius, and soleus muscle mass. Treatment with CSE significantly increased muscle mass in aged mice, particularly in the quadriceps and gastrocnemius muscles. The magnitude of recovery was comparable to that observed with oxymetholone treatment. Collectively, these findings demonstrate that CSE effectively attenuates both obesity- and aging-associated skeletal muscle loss.

**Table 2:** Effects of celery seed extract on skeletal muscle mass in aged mice.

g per 100 g body weight	YC	AC	CSE50	CSE100	CSE200	OXM
Quadriceps	0.98 ± 0.02	0.64 ± 0.02^a***^	0.77 ± 0.02^**b##**^	0.75 ± 0.03^**b#**^	0.74 ± 0.02^**b##**^	0.78 ± 0.04^**b#**^
Tibialis anterior	0.50 ± 0.001	0.33 ± 0.03^a**^	0.39 ± 0.01^**b**^	0.40 ± 0.01^**b**^	0.37 ± 0.01^**ab**^	0.38 ± 0.02^**b**^
Gastrocnemius	1.07 ± 0.02	0.76 ± 0.02^a***^	0.88 ± 0.02^**bc##**^	0.89 ± 0.02^**c##**^	0.82 ± 0.01^**ab#**^	0.80 ± 0.03^**a###**^
Soleus	0.058 ± 0.003	0.032 ± 0.002^a***^	0.049 ± 0.002^**b###**^	0.046 ± 0.002^**b###**^	0.045 ± 0.001^**b###**^	0.048 ± 0.002^**b###**^

Skeletal muscle mass is expressed as g per 100 g body weight. Data are presented as mean ± SEM (n = 6–8). ^**^P < 0.01, ^***^P < 0.001 versus YC; ^#^*P* < 0.05, ^##^*P* < 0.01, ^###^*P* < 0.001 versus AC. YC, young control; AC, aged control; CSE, celery seed extract (50, 100, or 200 mg/kg body weight); OXM, oxymetholone (50 mg/kg body weight). Different letters (a–c) indicate significant differences among the aged groups at *P* < 0.05.

### 3.2 CSE reduces intramuscular lipid accumulation and is associated with improved mitochondrial metabolism and AMPK–PGC-1α signaling

**Figure 2:**
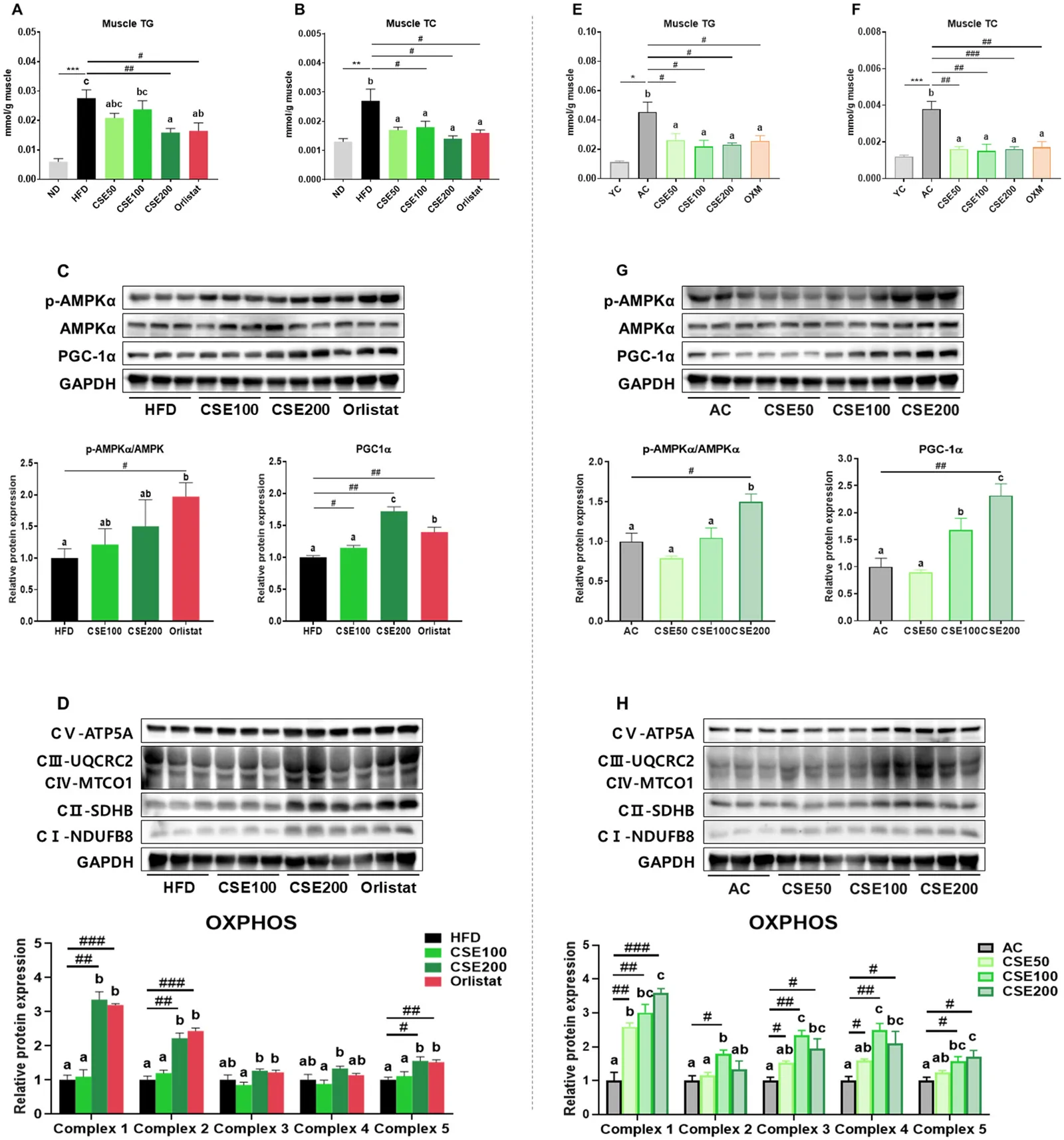
**Celery seed extract attenuates intramuscular lipid accumulation and is associated with changes in AMPK–PGC-1α** **signaling and mitochondrial metabolism-related protein expression in obese and aged mice. (A, E)** Muscle triglyceride (TG) levels. **(B, F)** Muscle total cholesterol (TC) levels. **(C, G)** Representative western blot images and quantification of p-AMPKα/AMPKα and PGC-1α protein expression in skeletal muscle (*n* = 3 per group). **(D, H)** Representative western blot images and quantification of mitochondrial oxidative phosphorylation (OXPHOS) complexes in skeletal muscle (*n* = 3 per group). Data are presented as mean ± SEM (*n* = 6–8). *P<*0.05*, **P<*0.01*, ***P<*0.001* versus ND or YC; ^#^*P*<*0.05*, ^*##*^*P*<*0.01*, ^*###*^*P*<*0.001* versus HFD or AC. ND, normal diet; HFD, high-fat diet; CSE, celery seed extract (50, 100, or 200 mg/kg body weight); Orlistat, 20 mg/kg body weight; YC, young control; AC, aged control; OXM, oxymetholone (50 mg/kg body weight). Different letters (a–c) indicate significant differences among the HFD or aged groups at *P*<*0.05*.

To investigate whether the protective effects of CSE on skeletal muscle were associated with alterations in intramuscular lipid metabolism and mitochondrial metabolism, TG and TC levels were measured in muscle tissues. In the HFD model, HFD feeding significantly increased muscle TG and TC levels compared with ND controls (Figures 2A, 2B). Treatment with CSE markedly reduced both TG and TC accumulation in skeletal muscle. A similar pattern was observed in the aging model. AC mice exhibited significantly elevated muscle TG and TC levels compared with young controls (Figures 2E, 2F), whereas CSE treatment significantly attenuated lipid accumulation in skeletal muscle.

Because intramuscular lipid overload is known to impair mitochondrial energy metabolism, we next examined the AMPK–PGC1α signaling pathway. In HFD-fed mice, CSE treatment increased AMPK phosphorylation and significantly upregulated PGC-1α protein expression compared with the HFD group (Figure 2C). Consistent with these findings, CSE administration also enhanced AMPK activation and PGC-1α expression in aged mice (Figure 2G). Given the critical role of PGC-1α in regulating mitochondrial biogenesis and oxidative metabolism, we further evaluated the expression of mitochondrial oxidative phosphorylation (OXPHOS) complexes in skeletal muscle. In the HFD model, CSE treatment increased the expression of several OXPHOS complex proteins compared with the HFD group (Figure 2D). Similar improvements were observed in aged mice, where CSE administration significantly elevated the expression of multiple OXPHOS complex proteins relative to the AC group (Figure 2H).

Together, these findings indicate that CSE alleviated intramuscular lipid accumulation and was associated with increased expression of mitochondrial metabolism-related proteins, accompanied by activation of the AMPK–PGC-1α signaling axis, in both obesity- and aging-associated muscle dysfunction.

### 3.3 CSE suppresses inflammatory signaling and NLRP3 inflammasome activation in skeletal muscle

To determine whether the beneficial effects of CSE on skeletal muscle were associated with modulation of inflammatory signaling, the expression of inflammatory mediators was evaluated in skeletal muscle tissues.

**Figure 3:**
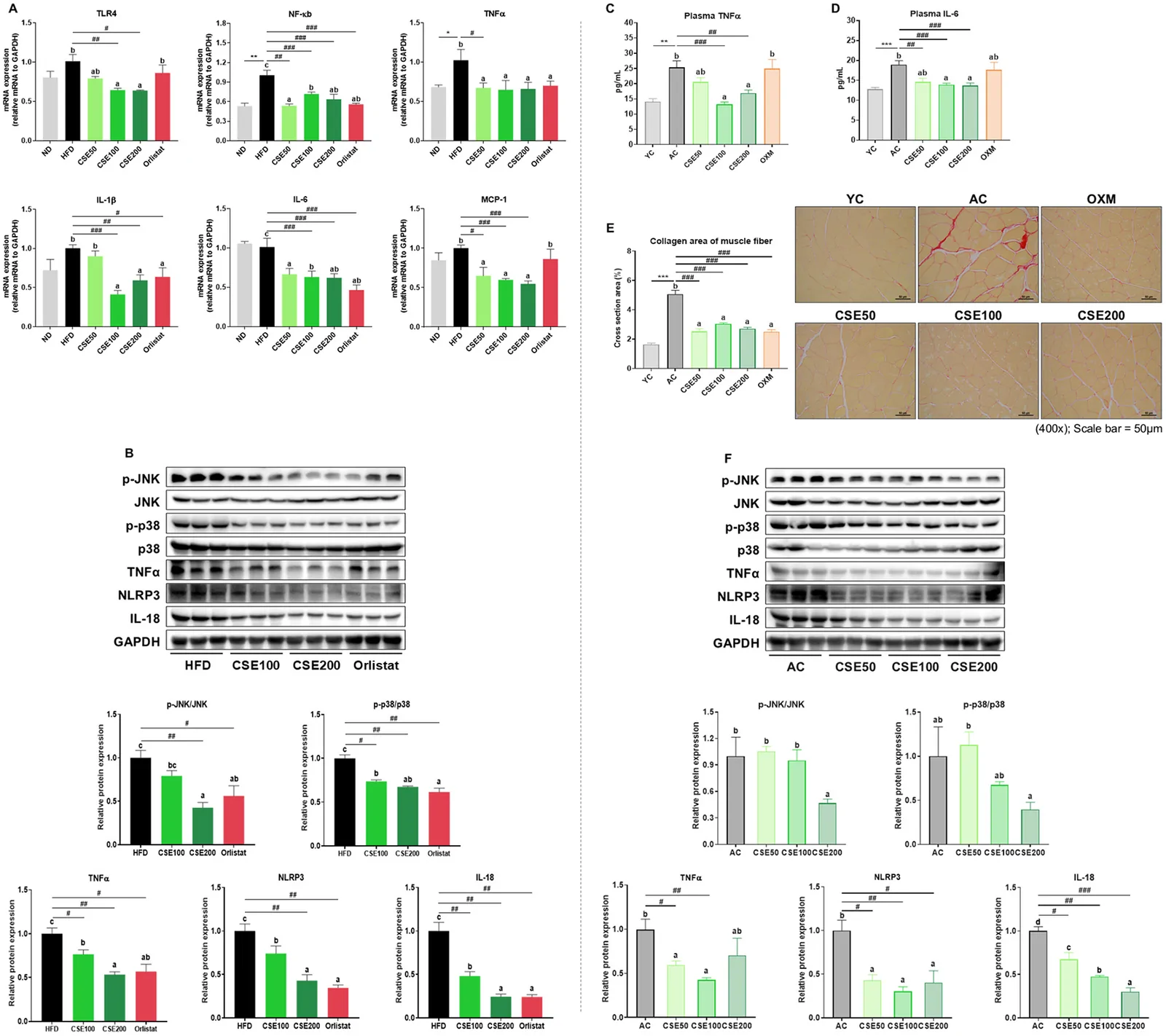
**Celery seed extract suppresses inflammatory signaling and NLRP3 inflammasome activation in obese and aged mice. (A)** Relative mRNA expression of inflammatory genes (TLR4, NF-κB, TNFα, IL-1β, IL-6, and MCP-1) in skeletal muscle of obese mice. **(B)** Representative western blot images and quantification of p-JNK/JNK, p-p38/p38, TNFα, NLRP3, and IL-18 protein expression in skeletal muscle (*n* = 3 per group). **(C)** Plasma TNFα levels in aged mice. **(D)** Plasma IL-6 levels in aged mice. **(E)** Representative Sirius Red–stained images of skeletal muscle and quantification of collagen area in aged mice. **(F)** Representative western blot images and quantification of p-JNK/JNK, p-p38/p38, TNFα, NLRP3, and IL-18 protein expression in skeletal muscle (*n* = 3 per group). Two consistently detected NLRP3-immunoreactive bands were quantified together for densitometric analysis. Data are presented as mean ± SEM (*n* = 6–8). *P<*0.05*, **P<*0.01*, ***P<*0.001* versus ND or YC; ^#^*P*<*0.05*, ^*##*^*P*<*0.01*, ^*###*^*P*<*0.001* versus HFD or AC. ND, normal diet; HFD, high-fat diet; CSE, celery seed extract (50, 100, or 200 mg/kg body weight); Orlistat, 20 mg/kg body weight; YC, young control; AC, aged control; OXM, oxymetholone (50 mg/kg body weight). Different letters (a–c) indicate significant differences among the HFD or aged groups at *P*<*0.05*.

In the HFD model, HFD feeding markedly increased the mRNA expression of multiple inflammatory markers, including toll-like receptor 4 (TLR4), nuclear factor kappa-light-chain-enhancer of activated B (NF-κB), TNF-α, IL-1β, IL-6, and monocyte Chemoattractant Protein-1 (MCP-1), compared with ND controls (Figure 3A). Administration of CSE significantly suppressed the expression of these inflammatory genes relative to the HFD group. Consistent with these transcriptional changes, activation of key inflammatory signaling pathways was also attenuated by CSE treatment. Western blot analysis revealed that HFD feeding increased phosphorylation of JNK and p38, accompanied by elevated expression of TNF-α, NLRP3, and IL-18 in skeletal muscle (Figure 3B). Treatment with CSE markedly reduced c-Jun N-terminal kinase (JNK) and p38 phosphorylation and decreased the expression of these inflammatory proteins.

A similar anti-inflammatory effect was observed in the aging model. Compared with young controls (YC), aged control (AC) mice exhibited significantly higher circulating levels of TNF-α and IL-6 (Figures 3C, 3D), indicating increased systemic inflammation. CSE administration significantly reduced plasma levels of these inflammatory cytokines. Histological analysis further demonstrated that aging promoted fibrotic remodeling in skeletal muscle. Aged mice showed increased collagen deposition surrounding muscle fibers, whereas CSE treatment markedly reduced collagen accumulation (Figure 3E). Consistent with these observations, aging also activated inflammatory signaling pathways in skeletal muscle. Aged control mice displayed increased phosphorylation of JNK and p38, along with elevated expression of TNF-α, NLRP3, and IL-18, whereas CSE treatment significantly suppressed these inflammatory responses (Figure 3F).

Collectively, these findings suggest that CSE alleviates both systemic and skeletal muscle inflammatory responses by inhibiting mitogen-activated protein kinases (MAPK)-dependent inflammatory signaling and NLRP3 inflammasome activation in obesity- and aging-associated muscle dysfunction.

### 3.4 CSE promotes myogenic signaling and suppresses proteolytic pathways in skeletal muscle

**Figure 4:**
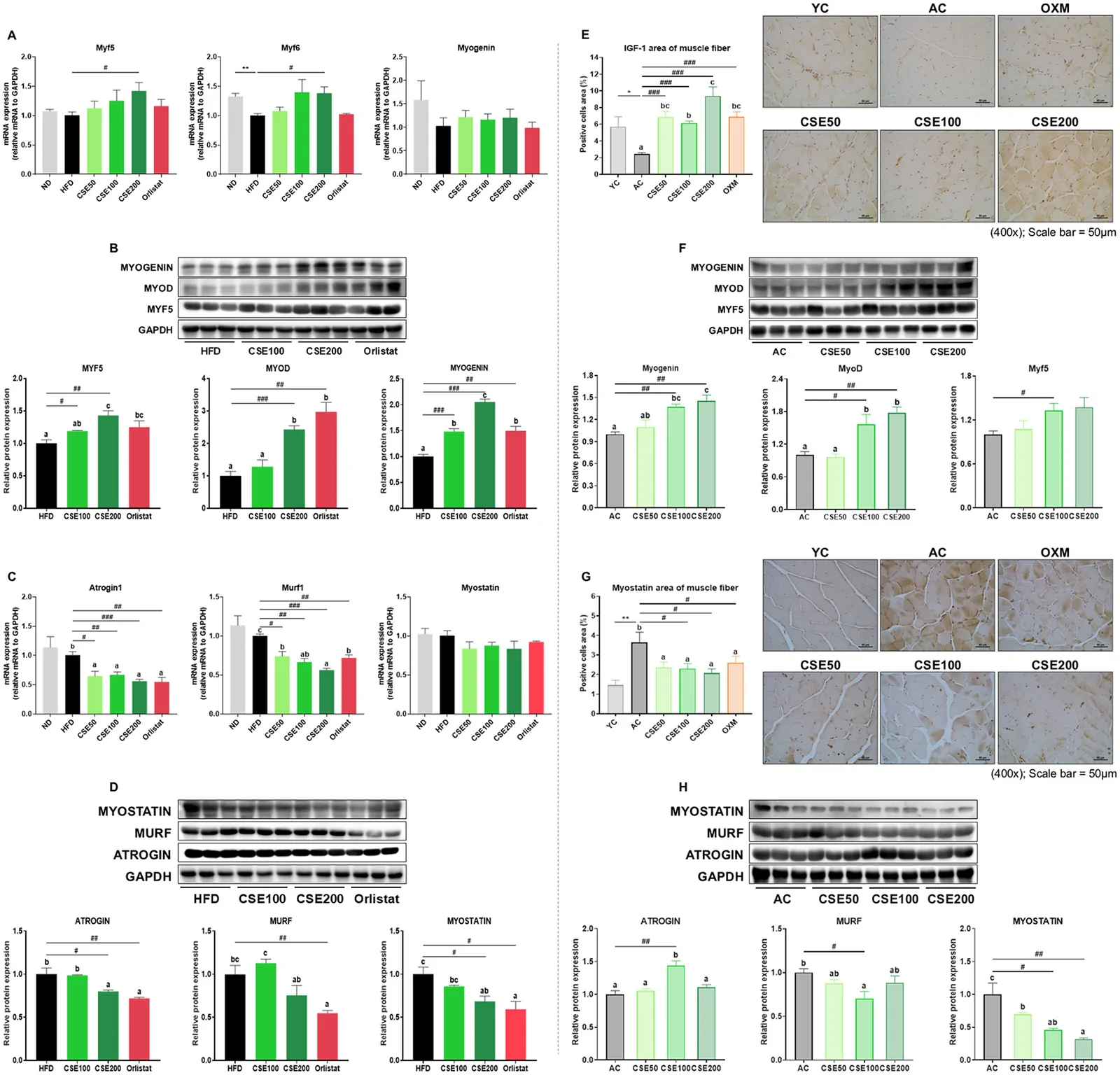
**Celery seed extract restores muscle protein homeostasis by enhancing myogenic signaling and suppressing proteolysis in obese and aged mice. (A)** Relative mRNA expression of myogenesis-related genes (Myf5, Myf6, and Myogenin) in skeletal muscle of obese mice. **(B)** Representative western blot images and quantification of Myogenin, MyoD, and Myf5 protein expression in skeletal muscle (*n* = 3 per group). **(C)** Relative mRNA expression of proteolysis-related genes (Atrogin-1, MuRF1, and Myostatin) in skeletal muscle of obese mice. **(D)** Representative western blot images and quantification of Myostatin, MuRF1, and Atrogin-1 protein expression in skeletal muscle (*n* = 3 per group). **(E)** Representative immunohistochemical staining and quantification of IGF-1–positive muscle fibers in skeletal muscle of aged mice. **(F)** Representative western blot images and quantification of Myogenin, MyoD, and Myf5 protein expression in skeletal muscle (*n* = 3 per group). **(G)** Representative immunohistochemical staining and quantification of Myostatin-positive muscle fibers in skeletal muscle of aged mice. **(H)** Representative western blot images and quantification of Myostatin, MuRF1, and Atrogin-1 protein expression in skeletal muscle (*n* = 3 per group). Data are presented as mean ± SEM (*n* = 6–8). *P<*0.05*, **P<*0.01*, ***P<*0.001* versus ND or YC; ^#^*P*<*0.05*, ^*##*^*P*<*0.01*, ^*###*^*P*<*0.001* versus HFD or AC. ND, normal diet; HFD, high-fat diet; CSE, celery seed extract (50, 100, or 200 mg/kg body weight); Orlistat, 20 mg/kg body weight; YC, young control; AC, aged control; OXM, oxymetholone (50 mg/kg body weight). Different letters (a–c) indicate significant differences among the HFD or aged groups at *P*<*0.05*.

To further investigate the mechanisms underlying the protective effects of CSE on skeletal muscle, we examined the expression of key regulators involved in myogenesis and muscle protein turnover. In the HFD model, HFD feeding reduced the expression of several myogenic markers, including myogenic factor 5 (Myf5), myogenic differentiation (MyoD), Myf6, and myogenin, compared with ND controls (Figures 4A, 4B). Treatment with CSE increased the expression of these myogenic factors, particularly Myf5, MyoD, and myogenin, indicating enhanced activation of myogenic signaling pathways. Consistent with these findings, CSE treatment also suppressed the expression of genes associated with muscle protein degradation. HFD feeding significantly increased the mRNA expression of Atrogin-1 and MuRF1, whereas CSE administration markedly reduced the expression of these atrophy-related genes (Figure 4C). Similar trends were observed at the protein level, where CSE treatment decreased the expression of myostatin, MuRF1, and Atrogin-1 compared with the HFD group (Figure 4D).

We next examined whether similar effects were observed in the aging model. Compared with YC mice, AC mice exhibited reduced myogenic activity, as evidenced by decreased expression of myogenin, MyoD, and Myf5 (Figure 4F). In addition, immunohistochemical analysis revealed that aging significantly reduced IGF-1–positive muscle fiber area, whereas CSE treatment restored IGF-1 expression within skeletal muscle (Figure 4E). Furthermore, aging was associated with increased myostatin accumulation within muscle fibers, indicating enhanced catabolic signaling. CSE administration markedly reduced myostatin-positive areas (Figure 4G). Consistently, Western blot analysis demonstrated that CSE treatment decreased the expression of Atrogin-1, MuRF1, and myostatin in aged skeletal muscle (Figure 4H).

Collectively, these findings suggest that CSE improves skeletal muscle homeostasis by promoting myogenic signaling while suppressing proteolytic pathways in both obesity- and aging-associated muscle dysfunction.

### 3.5 Intramuscular lipid accumulation is associated with mitochondrial dysfunction, inflammation, and muscle atrophy

**Figure 5:**
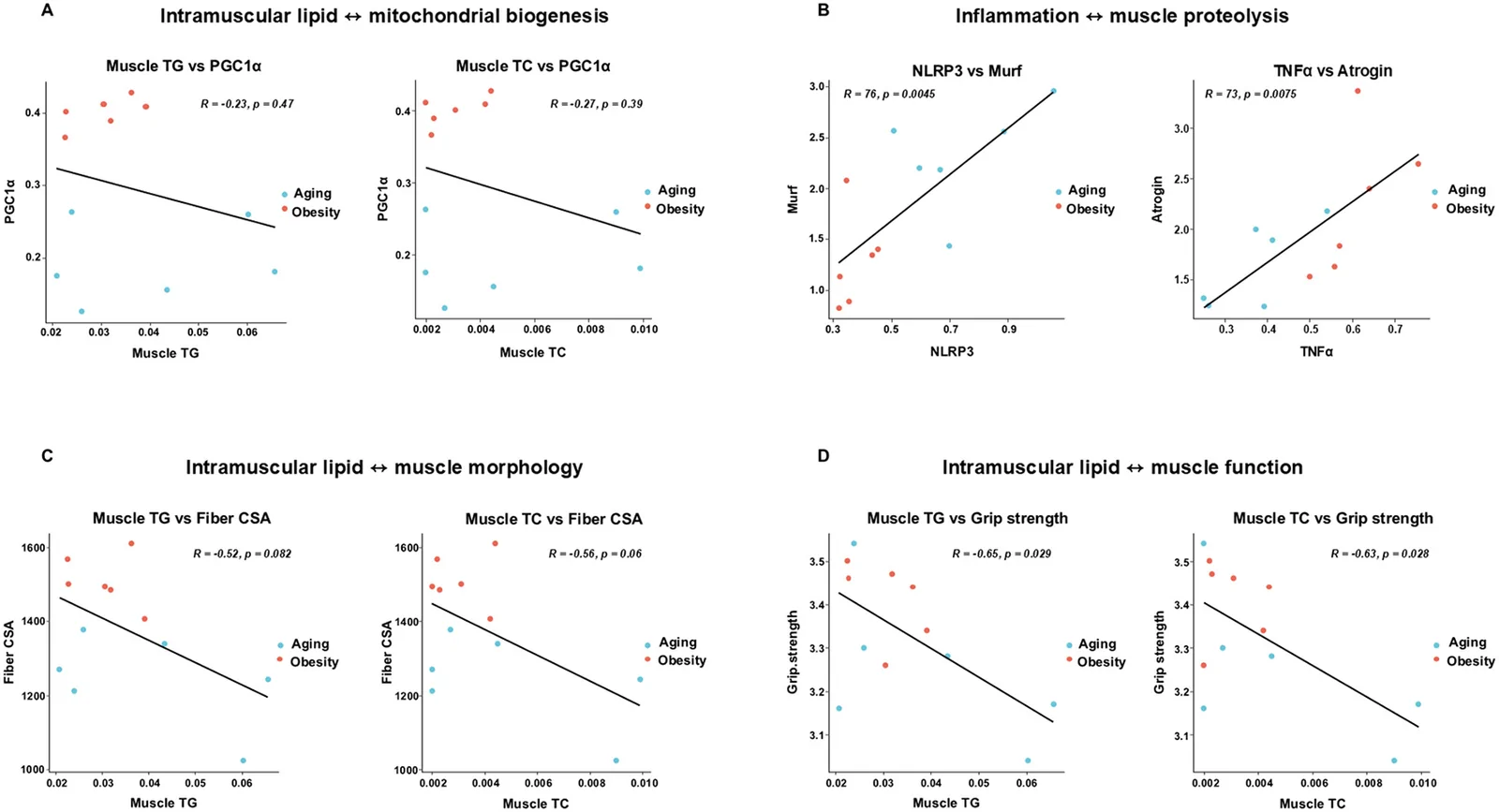
**Intramuscular lipid accumulation is associated with mitochondrial dysfunction, inflammatory activation, and muscle decline in obese and aged mice**. Correlation analyses were performed to examine the relationships among intramuscular lipid accumulation, inflammatory signaling, mitochondrial function, and muscle deterioration across obesity and aging models. **(A)** Correlations between intramuscular lipid levels (muscle TG and TC) and mitochondrial biogenesis marker PGC-1α. **(B)** Correlations between inflammatory signaling markers (NLRP3 and TNFα) and muscle proteolysis markers (MuRF-1 and Atrogin-1). **(C)** Correlations between intramuscular lipid levels and muscle fiber cross-sectional area (CSA). **(D)** Correlations between intramuscular lipid levels and muscle functional outcome (grip strength). Each dot represents an individual animal from the obesity or aging models. Pearson's correlation coefficients **(R)** and *p*-values are shown.

To further explore the relationships among intramuscular lipid accumulation, inflammatory signaling, and skeletal muscle dysfunction, correlation analyses were performed across samples from both the obesity and aging models. Intramuscular lipid levels were inversely associated with mitochondrial biogenesis–related signaling. Muscle TG and TC levels showed negative correlations with PGC-1α expression, suggesting that increased lipid accumulation in skeletal muscle is linked to impaired mitochondrial regulatory pathways (Figure 5A). In contrast, inflammatory signaling was positively associated with markers of muscle proteolysis. Expression of NLRP3 was positively correlated with MuRF1, and TNF-α levels were positively correlated with Atrogin-1, indicating that inflammatory activation is closely linked to enhanced muscle protein degradation (Figure 5B). We next examined whether intramuscular lipid accumulation was related to structural changes in skeletal muscle. Both muscle TG and TC levels showed inverse relationships with muscle fiber cross-sectional area, suggesting that increased lipid deposition is associated with reduced muscle fiber size (Figure 5C). Finally, intramuscular lipid accumulation was also negatively correlated with grip strength, indicating that elevated lipid content in skeletal muscle is associated with impaired muscle function (Figure 5D). Collectively, these findings highlight a close association between intramuscular lipid accumulation, mitochondrial dysfunction, inflammatory activation, and skeletal muscle structural and functional decline.

## 4 Discussion

The present study demonstrates that CSE attenuates skeletal muscle deterioration associated with both obesity and aging through modulation of intramuscular lipid accumulation and its downstream metabolic consequences. Using two complementary experimental models—diet-induced obesity and naturally aged mice—we consistently observed that CSE improved skeletal muscle mass, grip strength, muscle thickness, and muscle protein content, together with restoration of muscle fiber cross-sectional area. These findings indicate that CSE effectively ameliorates both structural and functional impairments of skeletal muscle that develop under metabolic and aging-related stress.

A key finding of the present study is that intramuscular lipid accumulation represents a shared metabolic feature linking obesity- and aging-associated skeletal muscle dysfunction. In both experimental models, increased lipid deposition within skeletal muscle was accompanied by impaired mitochondrial oxidative metabolism, activation of inflammatory signaling pathways, and dysregulation of muscle protein turnover. CSE supplementation markedly reduced intramuscular triglyceride and cholesterol accumulation and was associated with increased AMPK–PGC-1α signaling and expression of mitochondrial oxidative phosphorylation proteins. In parallel, CSE suppressed MAPK-dependent inflammatory signaling and NLRP3 inflammasome activation while improving muscle protein homeostasis by enhancing myogenic regulators and reducing the expression of atrophy-related factors. Correlation analyses further supported these relationships, demonstrating that intramuscular lipid accumulation was associated with reduced mitochondrial signaling, enhanced inflammatory activation, decreased muscle fiber size, and impaired muscle function. Collectively, these findings suggest that excessive intramuscular lipid accumulation may represent a common metabolic feature associated with mitochondrial metabolic impairment, inflammatory activation, and dysregulation of muscle protein turnover in both obesity- and aging-associated skeletal muscle dysfunction.

Accumulating evidence indicates that ectopic lipid accumulation within skeletal muscle, commonly referred to as intramuscular lipid accumulation, is a critical pathological feature connecting metabolic disturbances to skeletal muscle deterioration. Intramuscular lipid accumulation encompasses lipid deposition within muscle fibers as well as adipose infiltration between muscle bundles, and these lipid depots increase with aging and metabolic disorders such as obesity and type 2 diabetes (4). Increased fat infiltration in skeletal muscle has been associated with reduced muscle quality, impaired mobility, and increased risk of functional decline in older adults (5). Lipid infiltration can induce lipotoxic stress, mitochondrial dysfunction, oxidative stress, and inflammatory signaling, ultimately impairing muscle metabolism and promoting muscle loss (26). In addition, fibro-adipogenic progenitors within skeletal muscle may differentiate into adipocytes under pathological conditions, contributing to abnormal adipose deposition and muscle degeneration (27). Consistent with these observations, both obesity and aging in the present study were associated with increased skeletal muscle lipid accumulation, whereas CSE supplementation significantly reduced muscle lipid levels. These findings suggest that attenuation of intramuscular lipid accumulation represents an important mechanism through which CSE improves skeletal muscle structure and function.

Mitochondrial dysfunction is widely recognized as a major contributor to skeletal muscle deterioration during aging and metabolic disorders. Impaired mitochondrial oxidative metabolism disrupts energy homeostasis and contributes to muscle weakness and atrophy (10). The AMPK–PGC-1α signaling axis plays a central role in regulating mitochondrial biogenesis and oxidative metabolism (8, 9). PGC-1α coordinates mitochondrial gene expression through transcriptional regulators, including nuclear respiratory factors and mitochondrial transcription factor A, thereby promoting mitochondrial biogenesis and respiratory capacity (9, 28). Aging has been consistently associated with reductions in mitochondrial DNA abundance, oxidative enzyme activity, and ATP production in skeletal muscle (11). Importantly, excessive lipid accumulation within skeletal muscle can further aggravate mitochondrial dysfunction by suppressing fatty acid oxidation and increasing oxidative stress, thereby creating a vicious cycle that exacerbates metabolic impairment. Consistent with this mechanism, both obesity- and aging-associated models in the present study exhibited reduced AMPK activation, decreased PGC-1α expression, and impaired expression of mitochondrial oxidative phosphorylation complexes. CSE supplementation was associated with increased AMPK phosphorylation, increased PGC-1α expression, and increased expression of mitochondrial oxidative phosphorylation proteins. Collectively, these molecular changes are consistent with improved mitochondrial metabolic regulation, although direct functional assessment of mitochondrial bioenergetics will be required to confirm this interpretation.

Chronic low-grade inflammation is another key factor contributing to skeletal muscle deterioration during aging and metabolic disorders. Lipid accumulation within skeletal muscle can induce lipotoxic stress and activate inflammatory signaling pathways that disrupt muscle metabolic homeostasis (29). Among these pathways, activation of the NLRP3 inflammasome plays an important role in metabolic inflammation by promoting maturation of pro-inflammatory cytokines such as IL-1β and IL-18 (12). Increased expression of NLRP3 inflammasome components and inflammatory mediators has been observed in sarcopenic skeletal muscle and is associated with muscle fiber degeneration and functional decline (30). In addition, inflammatory cytokines such as IL-6 and TNF-α exert catabolic effects on skeletal muscle by inhibiting protein synthesis and activating proteolytic pathways, including the ubiquitin–proteasome system (16). Sustained elevation of IL-6 has also been shown to induce skeletal muscle atrophy (14, 31). Consistent with these findings, both obesity- and aging-associated models in the present study exhibited enhanced inflammatory signaling in skeletal muscle, including activation of MAPK pathways and increased expression of NLRP3 inflammasome components. These findings suggest that attenuation of lipotoxic inflammatory responses may be associated with the protective effects of CSE on skeletal muscle.

Maintenance of skeletal muscle mass depends on the balance between protein synthesis and protein degradation. Disruption of this balance toward increased proteolysis and impaired regeneration is a hallmark of sarcopenia. In skeletal muscle, anabolic pathways such as the IGF-1 axis promote protein synthesis and myogenic differentiation, whereas catabolic pathways mediated by ubiquitin–proteasome ligases promote protein degradation (32). IGF-1 stimulates expression of myogenic regulatory factors including MyoD and myogenin, which regulate satellite cell activation and muscle fiber regeneration (32, 33). Conversely, skeletal muscle atrophy is associated with activation of the ubiquitin–proteasome system, in which the E3 ubiquitin ligases Atrogin-1 and MuRF1 play central roles in muscle protein degradation (34). Myostatin acts as a potent negative regulator of muscle growth by suppressing protein synthesis and promoting proteolytic pathways (35). Obesity-related lipid accumulation and inflammation can further exacerbate this imbalance in muscle protein turnover (36). Consistent with these mechanisms, both obesity- and aging-associated models in the present study exhibited suppression of myogenic regulators together with activation of proteolytic signaling. These findings indicate that CSE was associated with enhanced anabolic signaling together with suppression of proteolytic pathways, consistent with improved skeletal muscle protein homeostasis.

The protective effects of CSE observed in the present study may be attributable, at least in part, to the diverse bioactive phytochemicals naturally present in celery seeds, including flavonoids, phenolic acids, and phthalides (40). Among these, flavonoids have attracted considerable attention because of their antioxidant and anti-inflammatory properties. In particular, apigenin, a representative flavonoid identified in celery seeds, has been shown to ameliorate obesity-associated skeletal muscle dysfunction by improving mitochondrial function and suppressing muscle catabolic signaling (38). In addition, apigenin has been reported to attenuate age-related muscle atrophy by reducing oxidative stress and preserving mitochondrial homeostasis (39). More recently, apigenin supplementation has also been shown to improve skeletal muscle dysfunction in obese middle-aged animals (37). These findings support the possibility that flavonoids present in celery seeds may contribute, at least in part, to the skeletal muscle-protective effects observed in the present study. Because the present study evaluated the biological efficacy of CSE as a whole extract rather than isolated phytochemicals, the observed effects are likely attributable to the complementary actions of multiple bioactive constituents rather than a single compound. Future studies employing comprehensive phytochemical profiling and bioactivity-guided fractionation will help clarify the relative contribution of individual constituents to the skeletal muscle-protective effects of CSE observed in the present study.

To place these findings within a broader metabolic context, transcriptomic analyses of liver and epididymal adipose tissue revealed alterations in pathways related to amino acid metabolism and nutrient-sensing processes under HFD conditions (Supplementary Figure 1). In particular, gene sets associated with branched-chain amino acid metabolism and translation-related pathways were enriched in metabolic tissues. Because adipose tissue plays an important role in regulating circulating amino acid availability and systemic insulin sensitivity, remodeling of amino acid metabolism in metabolic organs may influence systemic environments that regulate skeletal muscle protein homeostasis. Although these transcriptomic analyses were performed in metabolic tissues rather than skeletal muscle, they provide supportive evidence that systemic metabolic remodeling induced by CSE may contribute to the metabolic environment associated with skeletal muscle homeostasis.

Although obesity-associated sarcopenia and age-related sarcopenia differ in their initiating etiologies, the present study was designed based on the premise that excessive intramuscular lipid accumulation represents a common metabolic hallmark contributing to muscle dysfunction in both conditions. Therefore, our mechanistic investigation focused on pathways associated with intramuscular lipid metabolism and its downstream consequences, including mitochondrial dysfunction, inflammation, and impaired protein turnover, rather than disease-specific upstream signaling pathways. This approach enabled us to identify a common metabolic mechanism through which CSE exerts protective effects across both experimental models.

Finally, several limitations of this study should be acknowledged. First, the experiments were conducted using murine models of diet-induced obesity and natural aging, which may not fully recapitulate the complexity of human sarcopenia. Although obesity was confirmed by significant body weight gain following high-fat diet feeding, additional assessments of adiposity, including body fat mass, body fat percentage, and adipose tissue distribution, would have provided a more comprehensive characterization of the obesity phenotype. Furthermore, although the present study employed 55–56-week-old mice to model the early progression of age-associated muscle deterioration, this model represents a transitional stage between middle age and advanced aging rather than conventional late-stage sarcopenia. Therefore, caution should be exercised when extrapolating these findings to advanced sarcopenia in very old animals. Second, although strong associations were observed among intramuscular lipid accumulation, mitochondrial metabolism-related protein expression, inflammatory signaling, and muscle protein turnover, direct causal relationships among these pathways were not established. Third, transcriptomic analyses were limited to metabolic tissues under HFD conditions and therefore provide indirect evidence of systemic metabolic remodeling. Fourth, the present mechanistic analyses primarily focused on pathways related to intramuscular lipid metabolism, whereas other disease-specific signaling pathways, including mTOR-mediated anabolic signaling, were not investigated. Future studies integrating comprehensive assessments of adiposity, histological assessment of intramuscular lipid infiltration, direct functional analyses of mitochondrial bioenergetics, pathway-specific intervention experiments, and investigation of additional disease-specific signaling pathways will provide a more complete understanding of the protective mechanisms of CSE and facilitate translation of these findings to human populations.

## 5 Conclusion

In conclusion, CSE attenuates skeletal muscle deterioration in both obesity- and aging-associated sarcopenic conditions. CSE improved skeletal muscle mass, strength, and structural integrity while reducing intramuscular lipid accumulation. These protective effects were associated with improved mitochondrial metabolism-related signaling, accompanied by increased AMPK–PGC-1α signaling, suppression of lipotoxic inflammatory responses (including MAPK signaling and NLRP3 inflammasome activation), and rebalancing of muscle protein turnover through enhanced myogenic regulators and reduced proteolytic signaling. Importantly, our findings identify intramuscular lipid accumulation as a shared metabolic mechanism linking obesity and aging to skeletal muscle dysfunction. Collectively, this study provides mechanistic insight into the role of intramuscular lipid accumulation in sarcopenia and suggests that targeting intramuscular lipid metabolism may represent a promising nutritional strategy for mitigating skeletal muscle decline driven by metabolic and aging-related stress.

## Data Availability

The RNA-sequencing datasets generated in this study are available in the NCBI Sequence Read Archive (SRA) under BioProject accession number PRJNA1464358.
