# Supplementary material for: Celery seed extract attenuates sarcopenic obesity and age-related sarcopenia by reducing intramuscular lipid accumulation in mice
Source: Front Nutr. 2026 Sep 1;13:1861420. doi: 10.3389/fnut.2026.1861420 (PMC13576404; doi:10.3389/fnut.2026.1861420)
Supplement: Supplementary file 1 [file Supplementary_file_1.docx]

## **Supplementary Table 1.** Diet composition for animal experiment

| **Ingredient (g)** | **ND** | **HFD** |
| --- | --- | --- |
| Casein | 200.00 | 200.00 |
| D, L-methionine | 3.00 | 3.00 |
| Corn starch | 150.00 | 111.00 |
| Sucrose | 500.00 | 370.00 |
| Cellulose powder | 50.00 | 50.00 |
| Corn oil | 50.00 | 30.00 |
| Lard | — | 170.00 |
| Mineral mixture^1^ | 35.00 | 42.00 |
| Vitamin mixture^2^ | 10.00 | 12.00 |
| Choline bitartrate | 2.00 | 2.00 |
| Cholesterol | — | 10.00 |
| Tert-Butylhydroquinone | 0.01 | 0.04 |
| Total (g) | 1,000 | 1,000 |
| Total energy (kcal) | 3,850 | 4,524 |

ND, normal diet group; HFD, high-fat diet group. ^1^AIN-76 mineral mixture (grams/kg): calcium phosphate, 500; sodium chloride, 74; potassium citrate, 2220; potassium sulfate, 52; magnesium oxide, 24; manganous carbonate, 3.5; ferric citrate, 6; zinc carbonate, 1.6; cupric carbonate, 0.3; potassium iodate, 0.01; sodium celenite, 0.01; chromium potassium sulfate, 0.55; sucrose 118.03

^2^AIN-76 vitamin mixture (grams/kg): thiamin HCl, 0.6; riboflavin, 0.6; pyridoxine HCl, 0.7; nicotinic acid, 0.003; D-calcium pantothenate, 0.0016; folate, 0.2; D-biotin, 0.02; cyanocobalamin (vitamin B12), 0.001; retinyl palmitate premix, 0.8; DL-alpha tocopheryl acetate, premix, 20; cholecalciferol (vitamin D3), 0.0025; menaquinone (vitamin K), 0.05; antioxidant, 0.01; sucrose, finely powdered, 972.8

**Supplementary Table 2. Antibodies used in this study**

| **Primary antibody** | **Dilution factor** | **Company** | **Catalog No.** |
| --- | --- | --- | --- |
| AMPKα | 1:1000 | Cell Signaling Technology | 2532 |
| ATROGIN | 1:1000 | Abcam | ab168372 |
| IL-18 | 1:1000 | Cell Signaling Technology | 57058 |
| JNK | 1:1000 | Cell Signaling Technology | 9252 |
| GAPDH | 1:1000 | Cell Signaling Technology | 2118S |
| MYF5 | 1:1000 | Abcam | ab203076 |
| MYOD | 1:1000 | Invitrogen | MA1-41017 |
| MYOGENIN | 1:1000 | Abcam | ab124800 |
| MYOSTATIN | 1:1000 | Abcam | ab203076 |
| MURF | 1:1000 | Abcam | ab172479 |
| NLRP3 | 1:1000 | Cell Signaling Technology | 15101 |
| OXPHOS cocktail | 1:250 | Abcam | ab110413 |
| p-AMPKα | 1:1000 | Cell Signaling Technology | 2535 |
| PGC1α | 1:1000 | Abcam | ab54481 |
| P-JNK | 1:1000 | Cell Signaling Technology | 9251 |
| p38 | 1:500 | Cell Signaling Technology | 8690 |
| P-p38 | 1:500 | Cell Signaling Technology | 4511 |
| TNFα | 1:500 | Cell Signaling Technology | 11948 |
| **Secondary antibody** | **Dilution factor** | **Company** | **Catalog No.** |
| Anti-rabbit IgG | 1:1000 | Cell Signaling Technology | 7074S |
| Anti-mouse IgG | 1:1000 | Cell Signaling Technology | 7076S |

**Supplementary Table 3. Primer sequences used for RT-qPCR**

| **Gene name** | **Sequence (5’-3’)** |
| --- | --- |
| Atrogin-1 | F: AAC CGG GAG GCC AGC TAA AGA ACA  R: TGG GCC TAC AGA ACA GAC AGT GC |
| *Gapdh* | F: TGC AGT GGC AAA GTG GAG AT  R: TTG AAT TTG CCG TGA GTG GA |
| Myf6 | F: AA GAA AAT CTT GAG GGT GCG G  R: TTA GCC GTT ATC ACG AGC CC |
| IL-1β | F: TGA GCA CCT TCT TTT CCT TCA  R: TTG TCT AAT GGG AAC GTC ACA C |
| IL-6 | F: GAG GAT ACC ACT CCC AAC AGA CC  R: AAG TGC ATC ATC GTT GTT CAT ACA |
| MCP-1 | F: TTC CTC CAC CAC CAT GCA G  R: CCA GCC GGC AAC TGT GA |
| Murf1 | F: GAG AAC CTG GAG AAG CAG CT  R: CCG CGG TTG GTC CAG TAG |
| Myf5 | F: AGG AAA AGA AGC CCT GAA GC  R: GCA AAA AGA ACA GGC AGA GG |
| Myf6 | F: AA GAA AAT CTT GAG GGT GCG G  R: TTA GCC GTT ATC ACG AGC CC |
| Myogenin | F: CCT TGC TCA GCT CCC TCA  R: TGG GAG TTG CAT TCA CTG G |
| Myostatin | F: AAC CTT CCC AGG ACC AGG AGA A  R: GGC TTC AAA ATC GAC CGT GAG G |
| NF-κB | F: GTG CAG AAA GAA GAC ATT GAG GTG  R: AGG CTA GGG TCA GCG TAT GG |
| TLR4 | F: AAG AGC CGG AAG GTT ATT GTG  R: CCC ATT CCA GGT AGG TGT TTC |
| TNFα | F: AAA GAC ACC ATG AGC ACA GAA AGC  R: GCC ACA AGC AGG AAT GAG AAG AG |

**Supplementary Fig. 1. Enrichment of amino acid metabolism and proteostasis-related pathways in liver and epididymal adipose tissue under HFD conditions.** Gene set enrichment analysis (GSEA) was performed using bulk RNA-seq data derived from epididymal adipose tissue (A, C) and liver (B, D). (A, B) Enrichment of amino acid metabolism–related pathways, including branched-chain amino acid (BCAA) degradation and broader amino acid metabolic processes. (C) Enrichment of gene sets related to protein synthesis and anabolic signaling pathways, including translation, insulin signaling, and mTOR signaling. (D) Enrichment of gene sets associated with muscle atrophy–related pathways, including FOXO-mediated transcription and proteasome degradation. Enrichment is expressed as normalized enrichment scores (NES). Dot size represents statistical significance. Comparisons include HFD vs. ND, Orlistat vs. HFD, and CSE vs. HFD.
